# Spatiotemporal visualization for the global COVID-19 surveillance by balloon chart

**DOI:** 10.1186/s40249-021-00800-z

**Published:** 2021-03-01

**Authors:** Ming-Fan Pang, Zuo-Ru Liang, Zhi-Da Cheng, Xin-Ping Yang, Jie-Wen Wu, Ke Lyu, Jing-Jing Xi, Zhong-Jie Li, Guo-Qing Shi, Yan-Ping Zhang, George F. Gao, Xiao-Peng Qi, Xiao-Ping Dong

**Affiliations:** 1 Center for Global Public Health, Chinese Center for Disease Control and Prevention, Changping District, Beijing, 102206 China; 2 Yidu Cloud (Beijing) Technology Co., Ltd., Beijing, China

**Keywords:** COVID-19, Global, Surveillance, Spatiotemporal visualization, Balloon chart

## Abstract

**Background:**

Considering the widespread of coronavirus disease 2019 (COVID-19) pandemic in the world, it is important to understand the spatiotemporal development of the pandemic. In this study, we aimed to visualize time-associated alterations of COVID-19 in the context of continents and countries.

**Methods:**

Using COVID-19 case and death data from February to December 2020 offered by Johns Hopkins University, we generated time-associated balloon charts with multiple epidemiological indicators including crude case fatality rate (CFR), morbidity, mortality and the total number of cases, to compare the progression of the pandemic within a specific period across regions and countries, integrating seven related dimensions together. The area chart is used to supplement the display of the balloon chart in daily new COVID-19 case changes in UN geographic regions over time. Javascript and Vega-Lite were chosen for programming and mapping COVID-19 data in browsers for visualization.

**Results:**

From February 1st to December 20th 2020, the COVID-19 pandemic spread across UN subregions in the chronological order. It was first reported in East Asia, and then became noticeable in Europe (South, West and North), North America, East Europe and West Asia, Central and South America, Southern Africa, Caribbean, South Asia, North Africa, Southeast Asia and Oceania, causing several waves of epidemics in different regions. Since October, the balloons of Europe, North America and West Asia have been rising rapidly, reaching a dramatically high morbidity level ranging from 200 to 500/10 000 by December, suggesting an emerging winter wave of COVID-19 which was much bigger than the previous ones. By late December 2020, some European and American countries displayed a leading mortality as high as or over 100/100 000, represented by Belgium, Czechia, Spain, France, Italy, UK, Hungary, Bulgaria, Peru, USA, Argentina, Brazil, Chile and Mexico. The mortality of Iran was the highest in Asia (over 60/100 000), and that of South Africa topped in Africa (40/100 000). In the last 15 days, the CFRs of most countries were at low levels of less than 5%, while Mexico had exceptional high CFR close to 10%.

**Conclusions:**

We creatively used visualization integrating 7-dimensional epidemiologic and spatiotemporal indicators to assess the progression of COVID-19 pandemic in terms of transmissibility and severity. Such methodology allows public health workers and policy makers to understand the epidemics comparatively and flexibly. 
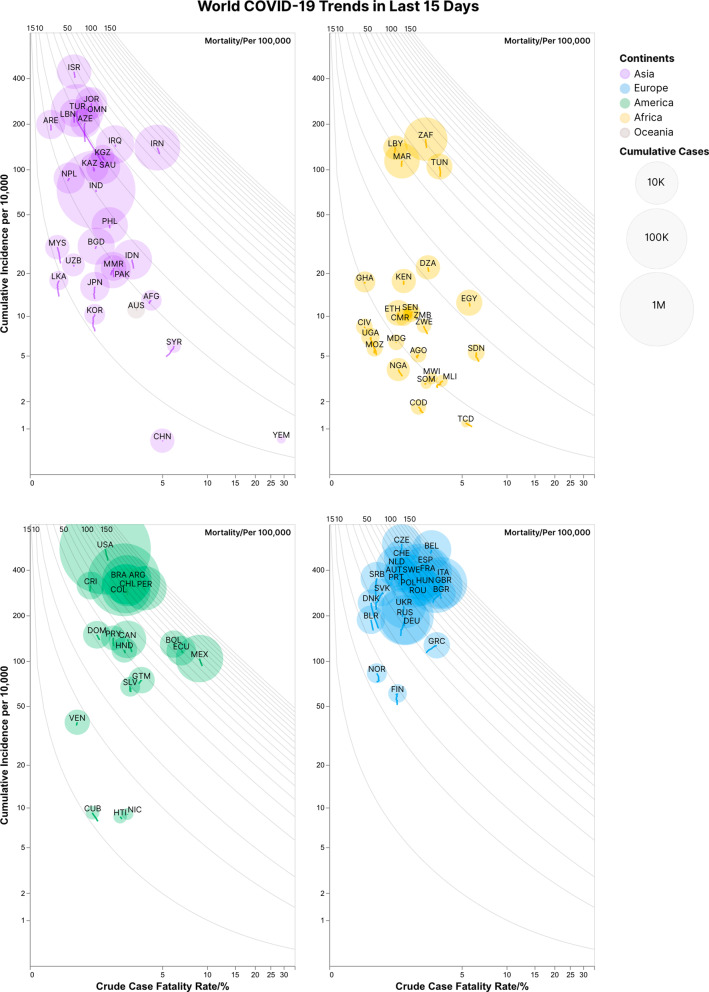

## Background

According to the World Health Organization (WHO), as of December 20th there have been over 75 million cases and over 1.6 million deaths of coronavirus disease 2019 (COVID-19) since the start of the pandemic [1]. Since the spread of the COVID-19 pandemic is still accelerating, with an extraordinary impact on global economies, social security and human lives, it is no longer a health issue but a social issue that inextricably links to everyone. Until now, there are bulk of open data published online for people to timely tracking the situation of the pandemic, however, only focus on the raw data is far from totally understanding the real situation and providing supportive evidence for decision making.

According to the Framework and Standards for Country Health Information Systems raised by WHO, as raw data, only through compilation, disposal, and analysis can data enhance its value to become information, which could then be used as supportive evidence for decision-making [2]. Visualization is a great way to transform raw data into valuable information, which can help people gain insights from complex data. Geographic visualization shows the geographical location and related information of all epidemic areas in an intuitive way; timeline visualization provides visualize temporal data in a specific period; spatiotemporal visualization combines geographical area and time interval to explore the deeper meaning of data. Those approaches are widely used in the COVID-19 pandemic, such as assessing the geographic risk of COVID-19 transmission [3], monitoring the control measures [4], presenting the timeline of COVID-19 outbreak [5], etc. Despite the variety of data visualization methods, for the pandemic disease like COVID-19, there is limited method to comprehensively demonstrate the global epidemic in one graph through constructing multi-dimensional core indicators.

In this paper, we provided a new methodology to visualize the spatiotemporal alteration of COVID-19 pandemic, presenting multi-dimensional information on one graph.

## Methods

### Data collection

All of the COVID-19 data were publicly available and were extracted from the COVID-19 data repository by the Center for Systems Science and Engineering at Johns Hopkins University [6]. The starting and ending date for the analysis were February 1 and December 20 respectively. According to the United Nations geographic regions [7], all countries were categorized into different continental regions, and then, subdivided into sub-regions, such as West Europe, East Europe, East Asia, West Asia, North Africa, etc.

### Construction of balloon charts and area charts

To evaluate the essential indicators reflecting the impact of COVID-19 pandemic on various continents, subregions and different countries, the “balloon chart” was generated. This innovation was based on a framework for assessing epidemiologic effects of influenza epidemics [8], and then improved by our team to assess the COVID-19 pandemic [9–11]. The three main epidemiological indicators in balloon chart included crude case fatality rate (CFR), cumulative incidence (CI, as a measure of morbidity) and mortality that were frequently used for assessment of the transmissibility and severity of an epidemic. CFR and CI were shown in X- and Y-axis, respectively. Mortality was illustrated in contour area separated with isolines. The cumulative number of COVID-19 cases was indicated with the size of the balloon. The colors of the balloons represented the different continents where the country or region is located. More importantly, the changing track of the COVID-19 epidemic of a region within a specific period was monitored with the movement of the small tail attached to the balloon.

As a supplement to balloon charts, area charts were used to illustrate the time-associated alteration of daily new cases in different continents and subregions. The 7-day moving average was calculated based on daily reported cases in order to eliminate the noise of data.

### Implementation of visualization

Vega-Lite [12] was used in this study for mapping the data to the properties of graphical marks. Compared to other tools, Vega-Lite shows a balance of flexibility and ease of configuration. Vega-Lite has been ported to different programming languages, and the native and most actively updated version is in JavaScript (d3 v6.0, vega-lite v4.17). We also choose JavaScript for the potential of interactive representation of data, and instant updating in browsers, allowing us to communicate and iterate the visualizations frequently.

## Results

### Time-associated alteration of the daily new cases of COVID-19 by geographic regions

**Fig. 1:**
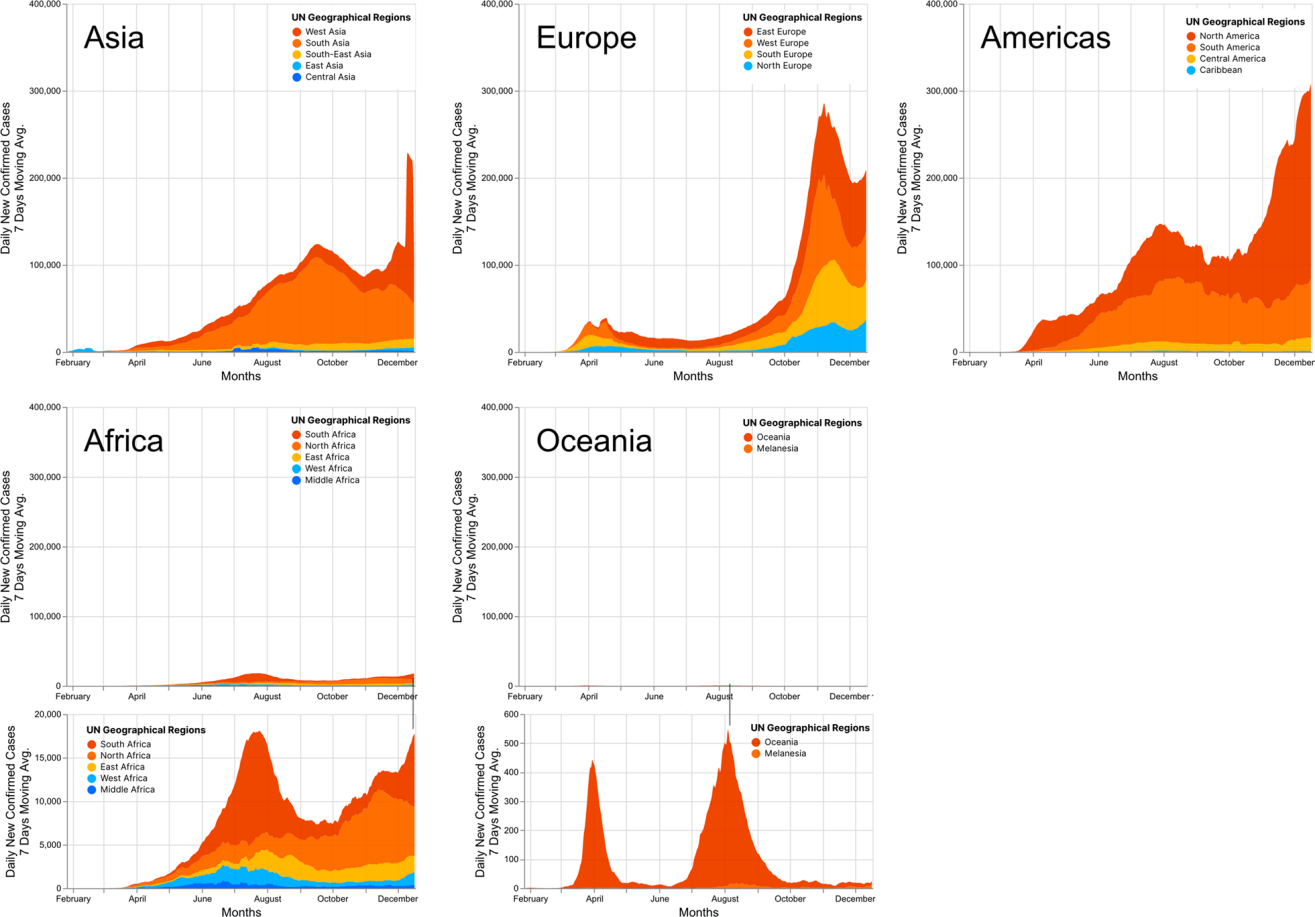
Time-associated alteration of the daily new confirmed COVID-19 cases by UN geographic region, Feb. 1st to Dec. 20th 2020. The 7-day moving average of daily reported COVID-19 cases was used to eliminate the noise of data. For Africa and Oceania, enlarged views were displayed

By December 20th 2020, most of the reported COVID-19 cases were in Asia, Europe and Americas. The distributions of daily COVID-19 cases varied largely among various continents and even in the subregions within continents along with the different times (Fig. 1). In Asia, the first wave appeared in East Asia (represented by China, Republic of Korea and Japan), which started in January 2020, reached the top in the middle of February and dropped down since the end of February. The second wave was much higher than the first one, which was mainly driven by cases from South Asia (represented by India) and West Asia (represented by Iran, Iraq and Turkey). It raised since the end of March and peaked mid-September with 120 000 new cases per day. After that, South Asia showed a gradual decrease in daily cases, while West Asia experience another increase starting from November, with a surge of reported cases on December 10 in Turkey. COVID-19 cases in the region of Southeast Asia showed a clear increase since July and maintained relatively stable afterwards. The case number of Central Asia and East Asia has been hovering at a low level with small fluctuation.

The first wave of daily COVID-19 cases in Europe raised since the beginning of March, reached the high plateau during April, and then slowly declined from late April to July. The second wave, seven times higher than the first one, started from August, peaked in early November with over 280 000 new cases per day, and then showed a sharp decrease in the same month. However, another relapse has appeared since December. The scale of daily cases in different subregions is similar to each other, while the starting time of the first wave in East Europe (represented by Russia, Ukraine and Poland) was one month later than the other subregions.

The daily cases in Americas started to increase rapidly since the middle of March and reached to the first peak at the beginning of August. The increase of new cases slowed down during August and September, and then started to rebound all the way up till now. At present, the daily cases has reached 300 000 per day, 70% of which was from North America (represented by the US and Canada) and 20% from South America (represented by Brazil, Peru and Chile). The daily cases in Central America and the Caribbean (represented by Mexico) reached a plateau in July and maintained relatively stable afterwards, despite of the fewer case numbers compared to North and South America.

The number of cases in Africa and Oceania was considerably lower than that of Asia, Europe and Americas. According to the enlarged view, the daily cases of Africa started to increase in mid-March, peaked around 17 000 cases/day in late July and then decreased. Since October, the daily cases has rebounded and hasn’t reached the plateau yet. The first wave was predominated by cases from Southern Africa (represented by South Africa), while the second one was largely contributed by cases from North Africa (represented by Morocco, Tunisia and Libya). Oceania (represented by Australia and New Zealand) had the smallest number of cases, which was no more than 600 cases/day during the two peaks in April and August. Since October the daily cases has remained below 50 cases/day.

### Time-associated alteration of the CFR, morbidity and mortality of COVID-19 in the context of the global geographic zones

**Fig. 2:**
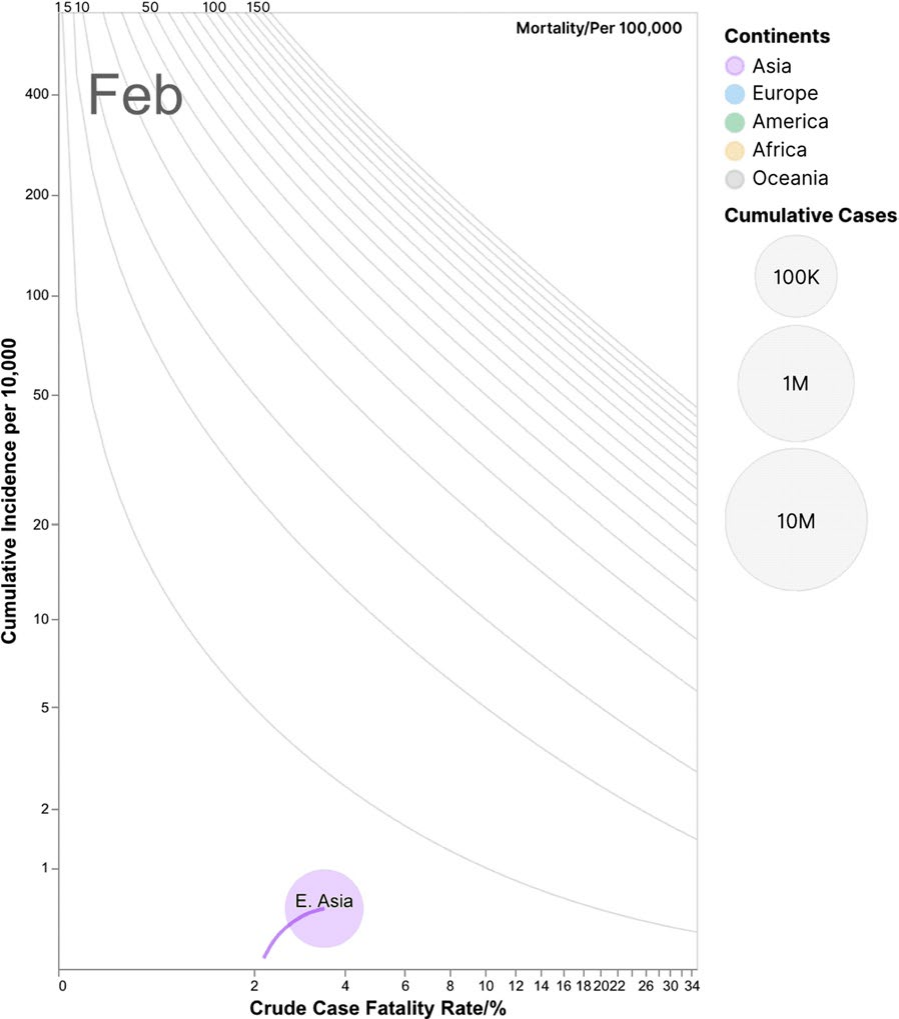
Time-associated alteration of the crude case-fatality rate, cumulative incidence and mortality of COVID-19 in UN geographic regions by month, Feb. 1st to Dec. 20th 2020. Cumulative incidence: Cumulative cases/population; CFR: Cumulative deaths/cumulative cases; Mortality: Cumulative deaths/population. The size of balloon represents cumulative number of confirmed COVID-19 cases. The tail of the balloon in each monthly freeze frame records the change of indicators in each region within the month. Regions with cumulative deaths less than 100 were not shown. X and Y-axis are plotted in log scale

In order to see the alterations of CFR, morbidity (or cumulative incidence) and mortality in the context of various geographic zones monthly, serial balloon charts from February to December were prepared as an animation (Fig. 2). The balloon tail in each monthly freeze frame represented the changing of COVID-19 in subregions within one month. In order to eliminate noise, we only showed subregions with cumulative deaths greater than 100 in each chart.

In February, only East Asia appeared in the graph within the zone of mortality below 1 person/100 000. In March and April, rapid increases of morbidity and CFR were identified in the South and West Europe (mortality over 30/100 000 by the end of April), as well as North Europe and North America (mortality around 20/100 000). The CFRs of South, North and West Europe were over 10%, higher than the rest of world. Broad regions in Asia, Europe, America and Africa started to report notable cases and deaths, whose mortality still below 5/100 000. West Asia, East Europe and South America experienced a rapid increase in morbidity, while Central America increased a lot in CFR.

From May to July, North and South America gradually exceeded Europe in morbidity, followed by Southern Africa, four regions in Europe, West Asia and Central America as the leading group by the end of July. Remarkable speed of case increase was observed in Southern Africa during this period, the cumulative incidence of which rose from one to 80 per 10 000. East Asia remained almost unchanged in morbidity. The rest of the regions worldwide experienced a rapid increase in the number of cases, especially Central Asia, South Asia and Caribbean. CFRs of most regions started to decrease in this period, except Central Asia.

From August to October, the balloons of the leading group kept going up in terms of morbidity, especially North and South Americas, the whole Europe, West and South Asia. CFRs of most leading regions decreased over time, except that of Southern Africa increasing slightly. Oceania experienced an obvious increase in CFR in August and September, and then stopped in October. In Africa, the morbidity of Southern Africa was as high as European regions, and that of North Africa increased rapidly, while that of the other parts was still low and changed little. Southeast Asia had a notable increase in morbidity during this period. East Asia still lied at the bottom.

In the recent two months, November and December, the balloons of North and South Americas, the whole Europe and West Asia rose dramatically in morbidity, followed by Southern Africa and Central America. The other regions experienced a slight increase in morbidity, with East Asia at the bottom. CFRs of all the regions worldwide either unchanged or decreased in this period. The CFR of Central America was obviously higher than other regions.

### Alteration of the CFR, morbidity and mortality of COVID-19 in the recent 15 days by continent and country

**Fig. 3:**
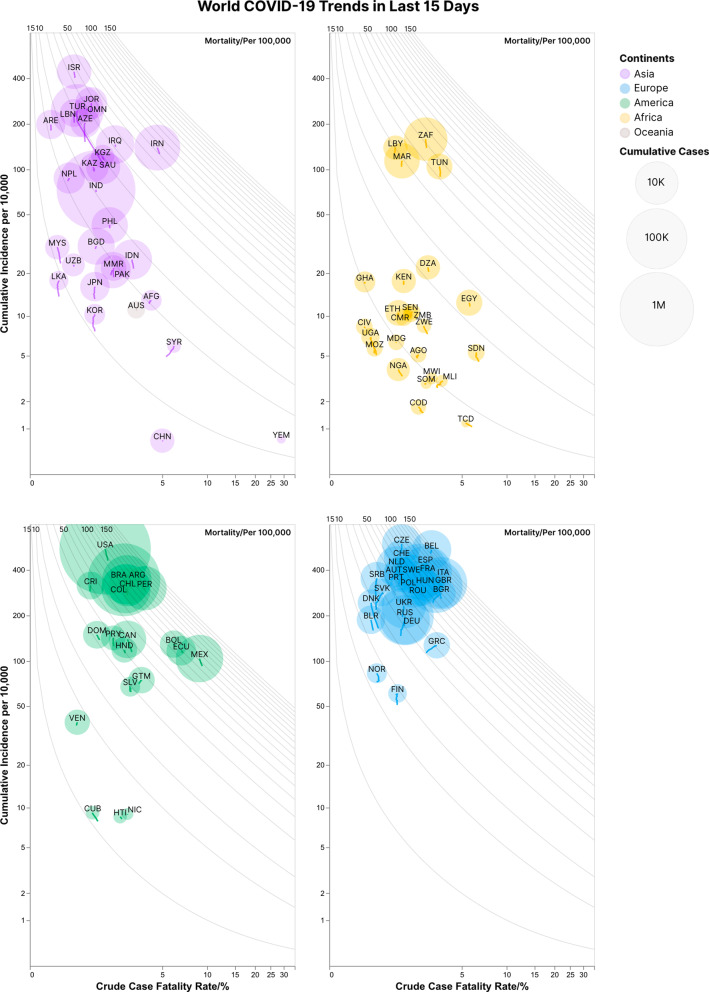
Alteration of the crude case-fatality rate, cumulative incidence and mortality of COVID-19 in last 15 days in December by country. Cumulative incidence: Cumulative cases/population; CFR: Cumulative deaths/cumulative cases; Mortality: Cumulative deaths/population. The size of balloon represents cumulative number of confirmed COVID-19 cases. The tail of the balloon records the change of indicators in each country within last 15 days ending on Dec. 24th. Only the countries with cumulative deaths more than 100 and population more than 5 million were shown. X and Y-axis are plotted in log scale. Country codes are as follows. Asia: AFG-Afghanistan, ARE-United Arab Emirates, AUS-Australia, AZE-Azerbaijan, BGD-Bangladesh, CHN-China, IDN-Indonesia, IND-India, IRN-Iran, IRQ-Iraq, ISR-Israel, JOR-Jordan, JPN-Japan, KAZ-Kazakhstan, KGZ-Kyrgyzstan, KOR-Republic of Korea, LBN-Lebanon, LKA-Sri Lanka, MMR-Burma, MYS-Malaysia, NPL-Nepal, OMN-Oman, PAK-Pakistan, PHL-Philippines, SAU-Saudi Arabia, SYR-Syria, TUR-Turkey, UZB-Uzbekistan, YEM-Yemen. Africa: AGO-Angola, CIV-Cote d'Ivoire, CMR-Cameroon, COD-Congo (Kinshasa), DZA-Algeria, EGY-Egypt, ETH-Ethiopia, GHA-Ghana, KEN-Kenya, LBY-Libya, MAR-Morocco, MDG-Madagascar, MLI-Mali, MOZ-Mozambique, MWI-Malawi, NGA-Nigeria, SDN-Sudan, SEN-Senegal, SOM-Somalia, TCD-Chad, TUN-Tunisia, UGA-Uganda, ZAF-South Africa, ZMB-Zambia, ZWE-Zimbabwe. America: ARG-Argentina, BOL-Bolivia, BRA-Brazil, CAN-Canada, CHL-Chile, COL-Colombia, CRI-Costa Rica, CUB-Cuba, DOM-Dominican Republic, ECU-Ecuador, GTM-Guatemala, HND-Honduras, HTI-Haiti, MEX-Mexico, NIC-Nicaragua, PER-Peru, PRY-Paraguay, SLV-El Salvador, USA-United States, VEN-Venezuela. Europe: AUT-Austria, BEL-Belgium, BGR-Bulgaria, BLR-Belarus, CHE-Switzerland, CZE-Czechia, DEU-Germany, DNK-Denmark, ESP-Spain, FIN-Finland, FRA-France, GBR-United Kingdom, GRC-Greece, HUN-Hungary, ITA-Italy, NLD-Netherlands, NOR-Norway, POL-Poland, PRT-Portugal, ROU-Romania, RUS-Russia, SRB-Serbia, SVK-Slovakia, SWE-Sweden, UKR-Ukraine

The changes of COVID-19 CFRs, morbidities and mortalities in the last 15 days of December were analyzed for countries with population larger than five million and COVID-19 cumulative deaths greater than 100. Countries were displayed by continent. As shown in Fig. 3, in the last 15 days of December, the overall mortalities of European and American countries were much higher than those of Asian and African countries.

In Asia, Iran had the highest mortality of over 60/100 000, followed by Israel, Jordan and Iraq locating at the zone of 30–40/100 000. Israel had the highest morbidity of more than 400/10 000. Turkey displayed a twice increase in morbidity, which was due to the surge of reported new cases on December 10. India had the largest number of cumulative cases, but in the recent days the increase of new cases has slowed down. China, Australia, the Philippines, Bangladesh and Uzbekistan maintained almost unchanged in position. The mortality of China was below 1/100 000, one of the lowest in the world. Iran and Syria had high CFRs of over 5%, and the CFR of Syria was still increasing. Yemen showed a remarkably high CFR of over 25%, despite of an obvious low morbidity.

In Africa, South Africa and Tunisia were in the mortality zone of 30–40/100 000, followed by Libya and Morocco with the mortality close to 20/100 000. The other African countries’ mortality was lower than 10/100 000. South Africa and Morocco had the largest number of cumulative cases up to now. The CFRs of Sudan, Egypt and Chad were over 5%, the highest in Africa in this period. Compared with other continents, Africa has fewer cases and no dramatic increase in morbidity or mortality.

In America, the United States had one of the highest morbidity and also the largest number of cases in the world up to now, and there was a rapid increase in the number of cases in the last 15 days. The mortality of the Peru, USA, Argentina, Brazil, Chile, Mexico, Colombia, Bolivia and Ecuador was in the range of 80–110/100 000, mainly driven by high morbidity except Mexico, Ecuador and Bolivia driven by high CFR. Costa Rica had a high morbidity, but its CFR was low. Mexico had the highest CFR of over 10%. The CFRs of all countries were either unchanged or decreasing. Cuba had the lowest mortality in America.

European countries had higher morbidity compared with other continents, ranging from 50 to 500 per 10 000, and all the countries had an increase in the number of cases in the past 15 days. Belgium had the highest mortality of 160/100 000 all over the world. Besides, many counties had a mortality close to or over 100/100 000, including Czechia, Spain, France, Italy, UK, Hungary and Bulgaria. The CFRs of the majority of countries changed little, while a few countries like Greece had a slight increase in CFR.

## Discussion

In this study, we used the balloon charts putting multiple epidemiologic indicators (i.e., morbidity, CFR and mortality) together in order to assess the progression of COVID-19 epidemic in terms of transmissibility and severity. Such methodology is more flexible to generate large or small-scale visible graph according to different geographic regions (e.g., continents or countries) and different epi-time (e.g., month or week), which allows the readers to analyze the spatiotemporal tracks of the epidemics comparatively. A tail attached to balloon is generated to pictorially record the changing track of the epidemic in a certain period, with the tail length representing the relative degree of change and the tail direction representing the dominating indicator. The chart successfully integrates multiple information into an intuitionistic visualization with epidemiological insight.

Using the balloon chart, we have drawn the monthly alterative graphs of UN subregions. Analysis and display according to this dimension can reflect the geographic spatial cluster of cases and the possible correlation with natural environmental factors. In the early stage of the outbreak, majority balloons of the subregions move apparently to the directions representing morbidity and CFR. After a certain period, the movement to CFR stopped or even retreated, meanwhile, the movement to morbidity was predominate and sizes of balloons became larger. Such phenomenon may reflect the comprehensive responses of the countries in the subregions to the COVID-19 epidemic, including implementation of containment or mitigation measures, enhancement of testing and therapeutic capacities, active case finding and management, etc. It is understandable that the national testing capacity in the early stage was usually low and lots of asymptomatic and mild cases were missed, contributing to a relatively high CFR.

Our monthly balloon charts presented the spreading of COVID-19 across various subregions and continents in a chronological order. Only the balloon of East Asia appeared on the chart in February. Afterwards from March to September, more balloons were noticed and became predominate in a time-associated manner: South Europe (represented by Italy and Spain), West and North Europe (Belgium, France, UK, Sweden), North America (the US and Canada), East Europe (Russia, Belarus) and West Asia (Iran, Iraq, Turkey), Central and South America (Brazil, Peru, Chile, Mexico), Southern Africa (South Africa), Caribbean, South Asia (India, Pakistan, Afghanistan), North Africa (Egypt and Morocco), Southeast Asia (Philippines and Indonesia) and Oceania (Australia). From May to July, the balloons of South, North and West Europe gradually stopped moving, reflecting that the epidemics in those regions were under control or remitted. At the same time, subregions in America, especially North and South America, exceeded Europe in morbidity and took the leading position thereafter. Since October, the balloons of Europe, North America and West Asia has been rising rapidly, reaching a dramatically high morbidity level by December, suggesting an emerging winter wave of COVID-19 which was much bigger than the previous ones. After a rapid increase in morbidity from May to September, Southern Africa and South Asia slowed down and has almost stopped moving in December. Although the balloons representing the regions of low incomes remained at the bottom area, including Africa (except Southern Africa) and Southeast Asia, we should be aware that there is lots of room for the balloons to go up if prevention and preparation interventions were absent or relaxed. Relatively undeveloped public health system and medical service in those regions would make it more difficult to control the COVID-19 pandemic in the future once these areas were severely affected. Compared with the traditional way of presenting geographical variation by maps, the balloon charts further allow readers to visualize the relative position of different regions without losing information about the quantitative measurement of transmissibility and severity. What’s more, it’s quite flexible for the balloon charts to display various dimension according to the outbreak scale and type of location, without relying on existing geocoded data, which it easier to use in resource poor countries.

The alteration features of represented countries from each continent in the last 15 days of December have been analyzed. Many European countries and the US showed obvious movement of their balloons to the direction of higher morbidity, reaching the mortality zone of 100/100 000 and above. As the coming of winter holidays and the emerging of SARS-CoV-2 variant recently identified in the UK [1], these countries need to take stronger measures and improve people’s compliance to contain the fast spreading of COVID-19. In Asia, Turkey had a long tail that doubled the number of cumulative cases, which was due to the surge on Dec. 10 caused by the adjustment of case definition to include mild cases [13]. In this period, the CFRs of most countries were at a low level of less than 5%, but Mexico had an exceptional high CFR close to 10% accompanying a high morbidity. As Mexico’s official said, the median age of death from COVID-19 was 55 years, much younger than an average of 75 years in many European countries, and the death pattern may result from high rates of underlying health conditions such as obesity, diabetes and hypertension [14]. China, the second populous country in the world and the first country reporting COVID-19 cases, still stayed in the lowest mortality zone by December. Active case finding with management [15, 16], and community-level public health promotion and interventions are China’s valuable experience to combat the pandemic [17].

In general, as of the end of December, different geographic locations around the world exhibited different epidemic characteristics. Although exploration of the reasons for such phenomenon is not the objective of the study here, display of spreading tracks and trends of the COVID-19 epidemic, regardless in large or small scales, will help us to understand the epi-features of this new infectious disease from another point of view.

The dynamic display of the balloon chart is more vivid and helpful for understanding of the pandemic. Monitoring the changes of indicators in various countries and regions in real time will provide decision support for the disease prevention and control. The balloon chart will be integrated in China CDC’s global infectious disease surveillance system in the future, allowing the balloon floating over time. Interactive browsing mode will be developed, and users can drag either the balloon or the timeline to compare the epidemiological indicators at different timepoints.

At present, the health information systems in many low and middle-income countries tend to be “data-rich” but “information-poor”. Our study proved that data cannot be used directly for decision-making without the value-added approach. We hope that through the widespread application of balloon charts, and the integration of more relevant information to create comprehensive visualization in the future, the value of data will be further realized.

The limitation of the study exists in the data quality of reported cases and deaths. When comparing the balloons of different countries and regions on one chart, the assumption is that the reporting timeliness and accuracy is similar across places. However, we only have access to the publicly available data on the internet. In the real world, the COVID-19 surveillance system may not function well in some places or during some time period, which will lead to under-estimation of the epidemic situation in these spatiotemporal intervals.

## Conclusions

We creatively used visualization integrating 7-dimensional epidemiologic and spatiotemporal indicators to assess the progression of COVID-19 pandemic in terms of transmissibility and severity. As of late December, the morbidity of Europe, North America and West Asia reached a dramatically high level and kept increasing, suggesting a developing winter wave of COVID-19 which was much bigger than the previous ones. Low-income countries in Africa and Southeast Asia showed low morbidity and mortality, but the situation could get worse if control measures were relaxed. It’s notable that Mexico still had an exceptional high CFR accompanying a high morbidity. China remained in the lowest mortality zone and its experience should be learned. Such methodology allows public health workers and policy makers to understand the epidemics comparatively and flexibly.

## Data Availability

The datasets used during the current study are available in the COVID-19 data repository from Johns Hopkins University, https://github.com/CSSEGISandData/COVID-19.
